# Foramen of Monro choroid plexus papilloma: An extremely rare location managed by endoscopic resection

**DOI:** 10.1016/j.radcr.2024.03.093

**Published:** 2024-05-03

**Authors:** Mahmoud Abdallat, Mohammed Aloqaily, Wafi Aloqaily, Mohammad Al-Qob, Saleh Al-Jbour, Abdallah Al-Muhtaseb, Nosaiba Al Ryalat, Fatima Obeidat

**Affiliations:** a Department of Neurosurgery, University of Jordan, Amman, Jordan; b Faculty of Medicine, The University of Jordan, Amman, Jordan

**Keywords:** Choroid plexus papilloma, Endoscopic resection, Foramen of Monro, Third ventricle

## Abstract

Choroid plexus papillomas are rare brain neoplasms, primarily observed in children, and typically manifest with symptoms indicative of heightened intracranial pressure and cerebral irritation. In addition, the tumor's localization varies with the patient's age, and diagnostic and therapeutic approaches predominantly rely on imaging findings and surgical interventions, with histopathological examination being essential for confirmation. This study outlines a unique instance of choroid plexus papilloma in a 30-year-old female, who presented with severe headache and vomiting, subsequently revealing hydrocephalus on Brain CT. Remarkably, the tumor was identified in the Foramen of Monro, an exceedingly rare and unreported location in adults. Notably, the patient underwent successful endoscopic resection without complications, a technique sparsely documented in similar cases. Choroid plexus papilloma, predominantly afflicting children, displays varied tumor locations depending on the patient's age. Our report highlights an exceptional case with an atypical tumor location that was not reported before to our knowledge, and addressed through an innovative endoscopic resection method that was recently used in the management of such cases. This underscores the importance of considering diverse tumor presentations, as it has a favorable prognosis achievable through management, especially with the increasing number of reported cases. Moreover, it advocates for the adoption of emerging endoscopic approaches, which exhibit promising outcomes.

## Introduction

Choroid plexus papilloma (CPP) is a rare brain tumor that is more commonly encountered in children and infants [1]. It composes around 4%-6% of brain tumors in pediatric populations in comparison to only 0.5% in adults [1]. Furthermore, it was noted that the tumor predilects toward certain locations based on age, as it is more common in lateral ventricles in children, meanwhile, in adults, it is more common to be found in the fourth ventricle [1,2].

The symptoms of increased intracranial pressure and hydrocephalus remain the most frequent presentation in most cases [1]. However, other neurological symptoms and focal neurological deficits can be the first manifestation, or they can be found incidentally [2,3]. The diagnosis of such tumors is done through imaging modalities such as computerized tomography (CT) and magnetic resonance imaging (MRI), although histopathological examination remains the cornerstone of definitive diagnosis, as imaging techniques are unable to differentiate between the different types of choroid plexus tumors or other types of brain tumors [4,5].

Herein, we present a case of a 30-year-old- female who was diagnosed with CPP in an unusual site and was managed through endoscopic total resection.

## Case presentation

A 30-year-old female with insignificant past medical history apart from glucose-6-phosphate deficiency (G6PD) and sulfa drug allergy, presented to the emergency medicine department complaining of severe headaches for 1 day. It was sudden, continuous, and affected the frontal area at the beginning, however, it became diffuse. The patient's headache was refractory to over-the-counter medications. Upon detailed asking, she denied any trauma history or other symptoms (such as blurry vision, dizziness, loss of consciousness, change in power, photophobia, etc.) apart from 2 vomiting episodes preceded by nausea (the first 3 days ago and the second in the Emergency department). It is worth mentioning that the patient's family said that she became forgetful about some daily events during the previous day.

On examination, her vital signs were stable, although she appeared in distress. The neurological exam revealed that she was conscious, alert, and oriented with a Glasgow coma scale (GCS) of 15/15. Furthermore, examination of the cerebellar, facial nerves, and limbs (for power, sensation, and reflexes) was unremarkable, and no focal neurological deficit was noticed. Nevertheless, the patient asked about the time several times during her stay in the emergency department which raised the suspicion of a hidden pathology. Other systemic examinations were normal, and no papilledema was found upon ophthalmological examination.

**Fig. 1:**
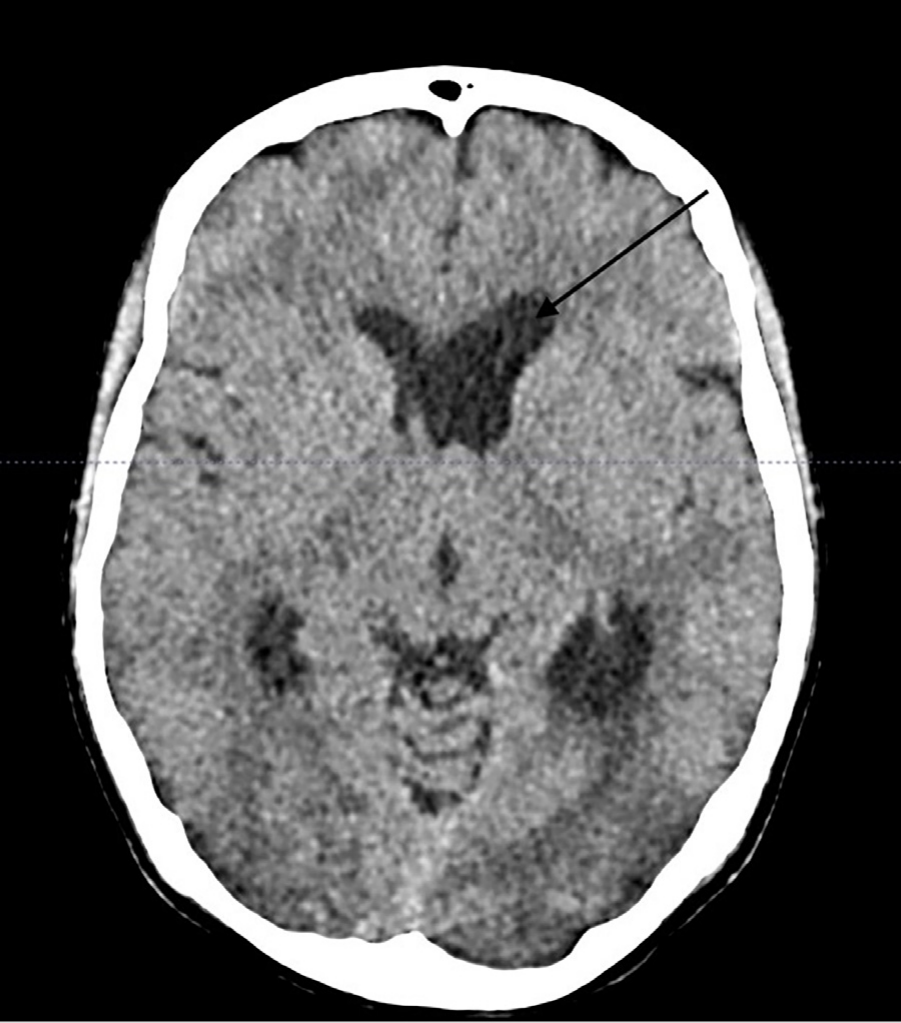
Pre-operative non-contrasted brain CT showing dilatation of the left lateral ventricle, suggesting obstructive unilateral hydrocephalus and suspicion of a small iso-dense lesion at the level of the left foramen of Monro.

The laboratory investigations did not reveal any significant abnormality. However, non-contrasted brain CT showed findings suggestive of obstructive unilateral hydrocephalus, Fig. 1.

**Fig. 2:**
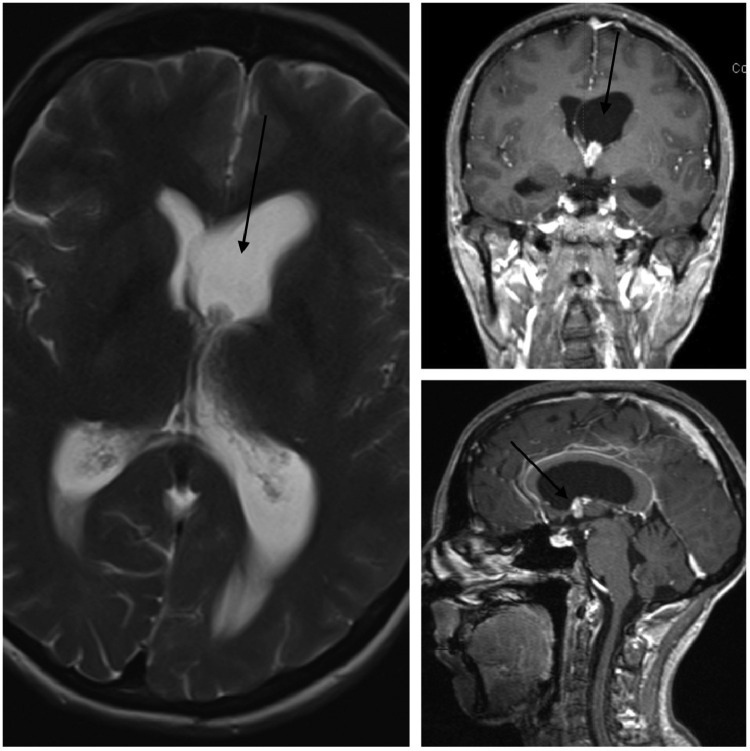
Pre-operative brain MRI with contrast exhibiting the presence of an avidly enhancing lesion at the left foramen of Monro protruding to the third ventricle roof with a lobulated, band-like enhancement adjacent to the lesion within the inferior portion of the left lateral ventricle (resembling continuation of the lesion).

Admission to the hospital for further evaluation was done. Upon admission, the contrasted brain MRI exhibited the presence of an avidly enhancing lesion at the left foramen of Monro protruding to the third ventricle roof, the lesion demonstrated a close relation to the adjacent choroid plexus papilloma which appeared engorged with a similar pattern of enhancement, Fig. 2. The lesion obstructed the left lateral ventricle, resulting in hydrocephalus; the findings were favored to represent a benign etiology. In addition, contrasted brain magnetic resonance venography (MRV) was negative for dural sinus thrombosis.

**Fig. 3:**
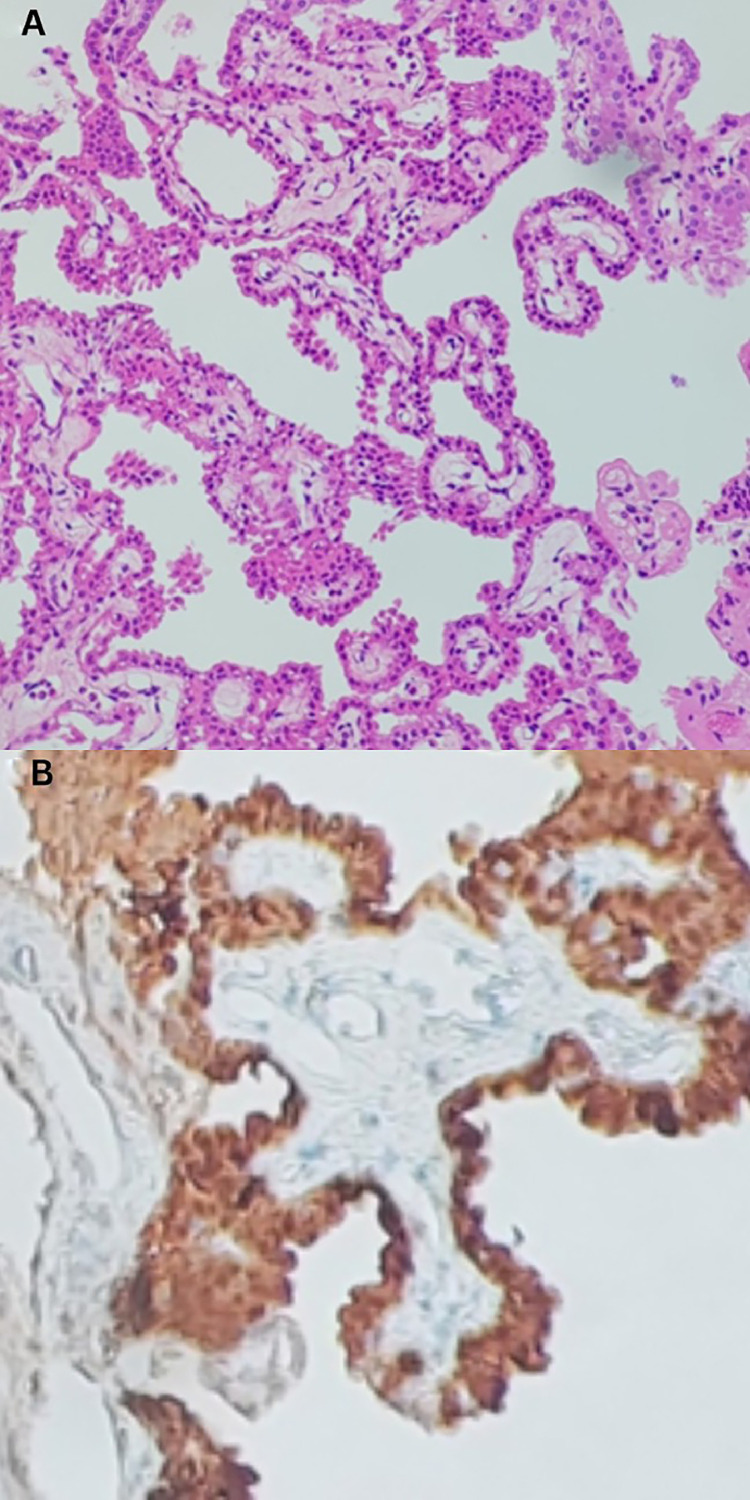
(A) Histologic section revealing papillary fronds lined by bland columnar epithelial cells without any mitotic activity, nuclear pleomorphism, or necrosis. (B) Columnar epithelial cells showing positive expression of S100 immunohistochemical stain.

**Fig. 4:**
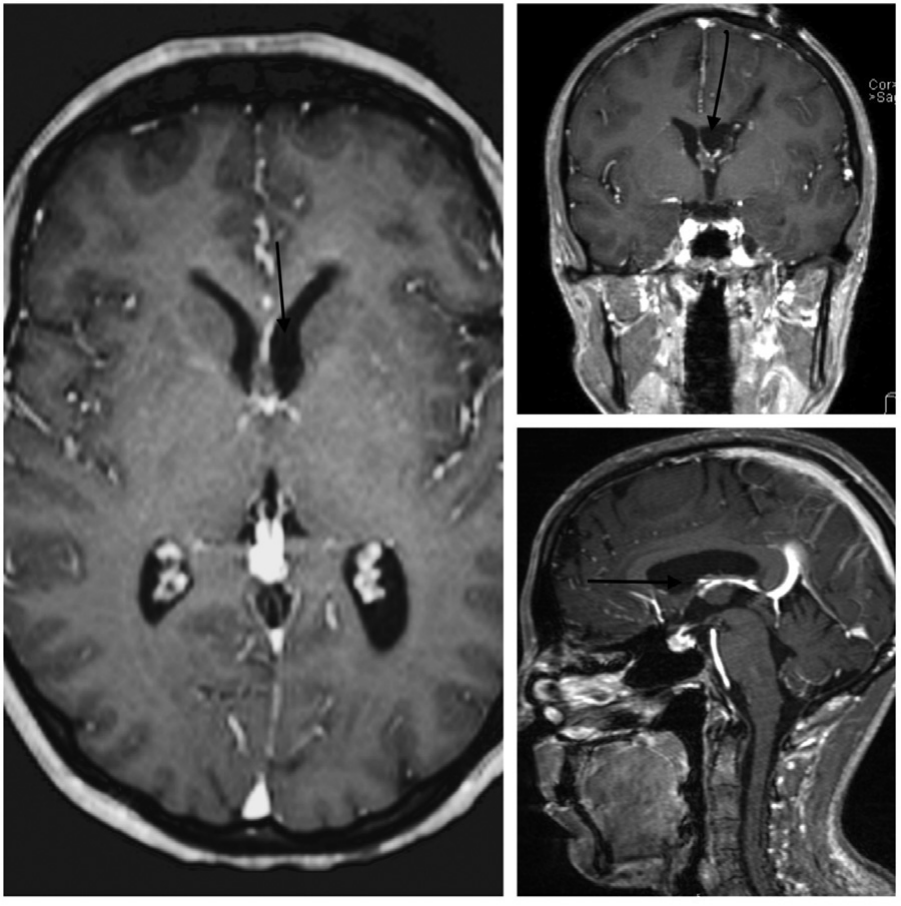
Post-operative brain MRI with contrast showing complete resection of the nodular enhancing lesion at the level of left foramen of monro without evidence of residual enhancement. Moreover, ventricular caliber improved considerably compared to pre-operative images.

Management options were discussed with the patient, and she decided to undergo the surgery. Accordingly, endoscopic tumor resection along with fenestration of septum pellucidum was done without intra-operative complications. The results of cerebrospinal fluid (CSF) analysis were (protein: 88.3, glucose 85.8, WBCs: 405 (78% PNL/22% lymphocytes), RBCs: 1696, and no bacterial growth). Several days later, the result of a brain biopsy unveiled the diagnosis of choroid plexus papilloma with calcifications, World Health Organization (WHO) grade 1, Fig. 3. The tumor was positive for S100 and focally positive for Pan cytokeratin (panCK). However, it was negative for Glial fibrillary acidic protein (GFAP) and Epithelial Membrane Antigen (EMA). Post-operative brain MRI **(**Fig. 4**)** showed complete resection of the tumor without residual enhancement. Moreover, the left ventricle was smaller in size, and no midline shift was noticed.

On follow-up, she complained of nausea and headache 4 days after the surgery (without any other symptoms), and the physical exam was unremarkable. Non-contrasted brain CT was done which was reassuring and did not show anything apart from evidence of previous surgery and improvement of the left ventricular dilation once compared to previous images. Furthermore, a follow-up brain MRI was done 3 months later and was unremarkable for any pathological changes.

## Discussion

Choroid plexus tumors (CPT) are considered rare tumors that originate from the choroid plexus epithelium. They were classified into three grades according to the newest World Health Organization (WHO); choroid plexus papilloma (CPP) (WHO grade 1), atypical choroid plexus papilloma (WHO grade 2), and choroid plexus carcinoma (CPC) (WHO grade 3), based on their mitotic activity [1,6].

Choroid plexus papilloma comprises less than 1% of brain tumors and even 0.5% percent of brain tumors in adults [1,3]. It is more common in males and can present at any age, with a higher predilection towards children [1,2]. Furthermore, it is of high importance to mention that the site of the tumor varies with the patient's age, as it tends to present mostly in the lateral ventricles (supratentorial area) in children, while in adults it is more common in the fourth ventricle (infratentorial area) [2,6]. Moreover, several cases of other rarer sites such as the cerebellopontine angle and third ventricle were reported in the literature. [5,6]. In this case, the tumor was located, surprisingly, in the left foramen of Monro and extended to the roof of the third ventricle, leading to unilateral hydrocephalus and resulting symptoms of ICP such as vomiting, which is of very rare occurrence and was not reported previously in the literature to our knowledge.

CPP can be found incidentally or present with various neurological symptoms such as headache, gaze, and vision problems, or a decrease in mental status, based on the location and size of the mass [2,3]. Moreover, symptoms of raised intracranial pressure and hydrocephalus remain frequently encountered, which is similar to the refractory headache and recent vomiting episodes in our patient [2,3]. Of notice, the hydrocephalus can develop either due to obstruction of the CSF flow or due to overproduction of the CSF from the tumor [1]. Moreover, it is worth mentioning that the development of CPP has been linked to various syndromes (Aicardi syndrome and Li–Li-Fraumeni syndrome), mutations (TP 53 mutation), and viruses (polyoma viruses family), which need more reports and studies to confirm such relations [2,7].

The diagnosis depends mainly on the findings of different imaging techniques such as CT and MRI, which will show iso- to hyperdense lesions on CT and hyperintense (lobulated or “cauliflower”) lesions on T2W1 MRI, along with the hydrocephalus and associated changes if present. The lesions mostly enhance on imaging, which is attributed to their high vascularity [1,2]. In addition, ultrasonographic modalities can be considered in younger patients if the fontanelles are open and as fast, safe, and non-invasive modalities to assess and exclude bleeding or vascular injuries [2,8]. Nevertheless, tumor biopsy and histopathological examination remain the “gold standard" and definitive diagnosis cannot be established without a biopsy as it diagnoses and differentiates between CPT subtypes [1], [2], [3]. Spinal MRI can be considered to evaluate for CSF seeding as cases were reported with both CPP and CPC, although much more common with CPC [9].

Total gross resection persists to be the cornerstone of management in symptomatic patients with a very high success rate and overall prognosis (over 95%) [2,9]. However, several other management methods have been studied recently to improve the results and to minimize the complications, such as radiotherapy, adjuvant chemotherapy, and tumor embolization (as CPT are highly vascular tumors with a high risk of bleeding that can be catastrophic, especially in infants and young patients) [2,10]. However, there is no consensus on whether to intervene or to follow-up if it is small and asymptomatic due to the very low risk of malignant transformation, although some cases were reported [2,7]. Furthermore, it is important to mention that endoscopic removal of such tumors was reported due to being less invasive and of help in special instances including the possibility of complications such as blood loss, mentioning it was used in the management of our case [6,11].

## Conclusion

Choroid plexus papilloma is a rare tumor that more commonly affects children rather than adults. The majority of cases present with symptoms of increased intracranial pressure and hydrocephalus. Current imaging techniques, such as CT and MRI, are not sufficient in distinguishing between different types of choroid plexus tumors or other brain tumors with similar characteristics. Therefore, histopathological evaluation is essential for accurate diagnosis. Moreover, the primary treatment for this tumor is complete removal, which has a high success rate and favorable prognosis with low recurrence. However, there is an increasing number of reported cases, and new management approaches, such as endoscopic removal, radiotherapy, and chemotherapy, are being explored to improve diagnostic accuracy and address associated complications.

## Patient consent

Consent for publication was taken from the patient.
